# Metabolites from *Alternaria* Fungi and Their Bioactivities

**DOI:** 10.3390/molecules18055891

**Published:** 2013-05-21

**Authors:** Jingfeng Lou, Linyun Fu, Youliang Peng, Ligang Zhou

**Affiliations:** MOA Key Laboratory of Plant Pathology, Department of Plant Pathology, College of Agronomy and Biotechnology, China Agricultural University, Beijing 100193, China

**Keywords:** metabolites, *Alternaria* fungi, biological activities, phytotoxins, mycotoxins, endophytes, plant pathogens

## Abstract

*Alternaria* is a cosmopolitan fungal genus widely distributing in soil and organic matter. It includes saprophytic, endophytic and pathogenic species. At least 268 metabolites from *Alternaria* fungi have been reported in the past few decades. They mainly include nitrogen-containing metabolites, steroids, terpenoids, pyranones, quinones, and phenolics. This review aims to briefly summarize the structurally different metabolites produced by *Alternaria* fungi, as well as their occurrences, biological activities and functions. Some considerations related to synthesis, biosynthesis, production and applications of the metabolites from *Alternaria* fungi are also discussed.

## 1. Introduction

*Alternaria* fungi, belonging to the Dematiaceae of the Hyphomycetes in the Fungi Imperfecti, have a widespread distribution in Nature. They act as plant pathogens, weak facultative parasites, saprophytes and endophytes [1]. Some metabolites from *Alternaria* fungi are toxic to plants and animals, and are designated as phytotoxins and mycotoxins, respectively [2,3,4]. *Alternaria* metabolites exhibit a variety of biological activities such as phytotoxic, cytotoxic, and antimicrobial properties, which have drawn the attention of many chemists, pharmacologists, and plant pathologists in research programs as well as in application studies [5,6]. For examples, porritoxin (**21**, Table 1) from endophytic *Alternaria* species has been studied as the candidate of cancer chemoproventive agent [7]. Depudecin (**257**), an inhibitor of histone deacetylase (HDAC) from *A. brassicicola*, also showed its antitumor potency [8,9]. Some *Alternaria* metabolites such as tenuazonic acid (**15**), maculosin (**43**) and tentoxin (**53**) have been studied as the herbicide candidates [10,11,12].

In the early 1990s, about 70 metabolites from *Alternaria* fungi were reviewed [13]. Several reviews on *Alternaria* phytotoxins have been published over the last few decades [6,14,15]. In recent years, more and more metabolites with bioactivities from *Alternaria* fungi have been isolated and structurally characterized. This review mainly presents classification, occurrences, biological activities and functions of the metabolites from *Alternaria* fungi. We also discussed and prospected the synthesis, biosynthesis, production and applications of the metabolites from *Alternaria* fungi.

## 2. Classification and Occurrence

The metabolites from *Alternaria* fungi can be grouped into several categories which include nitrogen-containing compounds, steroids, terpenoids, pyranones (pyrones), quinones, phenolics, *etc.* Several metabolites are unique to one *Alternaria* species, but most metabolites are produced by more than one species. Occurrences of the isolated metabolites from *Alternaria* fungi are listed in Table 1 [16,17,18,19,20,21,22,23,24,25,26,27,28,29,30,31,32,33,34,35,36,37,38,39,40,41,42,43,44,45,46,47,48,49,50,51,52,53,54,55,56,57,58,59,60,61,62,63,64,65,66,67,68,69,70,71,72,73,74,75,76,77,78,79,80,81,82,83,84,85,86,87,88,89,90,91,92,93,94,95,96,97,98,99,100,101,102,103,104,105,106,107,108,109,110,111,112,113,114,115,116,117,118,119,120,121,122,123,124,125,126,127,128,129,130,131,132,133,134,135]. The most widespread metabolite is alternariol (**157**) which has been isolated from a few *Alternaria* fungi [25,27,84,85]. Some metabolites were also isolated from other genus fungi and even from higher plants. Typical examples included AAL toxins **3**–**10** from *Fusarium* species [5,136,137], helvolic acid (**117**) from *Aspergillus* species [138] and *Pichia* species [139], paclitaxel (taxol, **61**) from yew trees (*Taxus* spp.) [140], resveratrol (**252**) from a variety of plant species such as *Vitis vinifera*, *Polygonum cuspidatum* and *Glycine max* [141], besides these metabolites from *Alternaria* species [43,53,130].

**Table 1:** The isolated metabolites and their occurrences in *Alternaria* fungi.

Metabolite class	Metabolite name	*Alternaria* species	Reference
Nitrogen-containing Metabolites	AAL-toxin TA_1_ (**1**)	*A. alternata* f.sp. *lycopersici*	[16,17]
	AAL-toxin TA_2_ (**2**)	*A. alternata* f.sp. *lycopersici*	[16,17]
	AAL-toxin TB_1_ (**3**)	*A. alternata* f.sp. *lycopersici*	[16,17]
	AAL-toxin TB_2_ (**4**)	*A. alternata* f.sp. *lycopersici*	[16,17]
	AAL-toxin TC_1_ (**5**)	*A. alternata* f.sp. *lycopersici*	[18]
	AAL-toxin TC_2_ (**6**)	*A. alternata* f.sp. *lycopersici*	[18]
	AAL-toxin TD_1_ (**7**)	*A. alternata* f.sp. *lycopersici*	[18]
	AAL-toxin TD_2_ (**8**)	*A. alternata* f.sp. *lycopersici*	[18]
	AAL-toxin TE_1_ (**9**)	*A. alternata* f.sp. *lycopersici*	[18]
	AAL-toxin TE_2_ (**10**)	*A. alternata* f.sp. *lycopersici*	[18]
	Fumonisin B_1_ (**11**)	*A. alternata*	[19]
		*A. alternata* f.sp. *lycopersici*	[20]
	Altersetin (**12**)	*Alternaria* sp.	[21]
	*N*-Acetyltyramine (**13**)	*A. tenuissima*	[22]
	Pyrophen (**14**)	*A. alternata*	[23]
	Tenuazonic acid = TeA = TA = AAC-toxin (**15**)	*A. alternata*	[24,25,26,27,28]
		*A. citri*	[29]
		*A, crassa*	[30]
		*A. linicola*	[31]
		*A. tenuissima*	[24]
	Deprenylzinnimide (**16**)	*A. porri*	[32]
	Zinnimide (**17**)	*A. porri*	[32]
	Cichorine (**18**)	*A. cichorii*	[33]
	Zinnimidine (**19**)	*A. cichorii*	[33]
		*A. porri*	[32,34,35]
	*Z*-Hydroxyzinnimidine (**20**)	*A. cichorii*	[33]
	Porritoxin (**21**)	*A. porri*	[7,36]
	Porritoxin sulfonic acid (**22**)	*A. porri*	[35]
	ACT-toxin I (**23**)	*A. alternata*	[37,38]
	ACT-toxin II (**24**)	*A. alternata*	[37,38]
	AK-toxin I (**25**)	*A. kikuchiana* (*A. alternata*)	[39]
	AK-toxin II (**26**)	*A. kikuchiana* (*A. alternata*)	[39]
	AS-I toxin (**27**)	*A. alternata*	[40]
	(2S,3S,4R,2'R)-2-(2'-hydroxytetracosanoylamino) Octadecane-1,3,4-triol (**28**)	*Alternaria* sp.	[41]
	Cerebroside B (**29**)	*Alternaria* sp.	[41]
	Cerebroside C (**30**)	*Alternaria* sp.	[41]
	AI-77-B (**31**)	*A. tenuis*	[42]
	AI-77-F (**32**)	*A. tenuis*	[42]
	Sg17-1-4 (**33**)	*A. tenuis*	[42]
	Cyclo-(Pro-Ala-) (**34**)	*A. alternata*	[10]
		*A. tenuissima*	[22]
Nitrogen-containing Metabolites	Cyclo-(Pro-Pro-) (**35**)	*A. tenuissima*	[22]
	Cyclo-(Phe-Ser-) (**36**)	*Alternaria* sp.FL25	[43]
	Cyclo-(l-Leu-*trans*-4-hydroxy-l-Pro-) (**37**)	*A. alternata*	[44]
		*A. tenuissima*	[22]
	Cyclo-(S-Pro-R-Val-) (**38**)	*A. alternata*	[10]
		*A. tenuissima*	[22]
	Cyclo-(Pro-Leu-) (**39**)	*A. tenuissima*	[22]
	Cyclo-(Pro-Homoleucine-) (**40**)	*A. alternata*	[10]
	Cyclo-(S-Pro-R-Ile-) (**41**)	*A. tenuissima*	[22]
	Cyclo-(Pro-Phe-) (**42**)	*A. alternata*	[10]
		*A. tenuissima*	[22]
	Maculosin = Cyclo-(l-Pro-l-Tyr-) (**43**)	*A. alternata*	[10]
	Cyclo-(l-Phe-*trans*-4-hydroxy-l-Pro-) (**44**)	*A. alternata*	[44]
	Cyclo-(l-Ala-*trans*-4-hydroxy-L-Pro-) (**45**)	*A. alternata*	[44]
	AM-toxin I (**46**)	*A. mali* (*A. alternata*)	[39]
	AM-toxin II (**47**)	*A. mali* (*A. alternata*)	[39]
	AM-toxin III (**48**)	*A. mali* (*A. alternata*)	[39]
	Destruxin A (**49**)	*A. linicola*	[31]
	Destruxin B (**50**)	*A. brassicae*	[45]
		*A. linicola*	[31]
	Homodestruxin B (**51**)	*A. brassicae*	[46]
	Desmethyldestruxin B (**52**)	*A. brassicae*	[46]
	Tentoxin (**53**)	*A. alternata*	[47]
		*A. citri*	[29]
		*A. linicola*	[31]
		*A. porri*	[48]
	Isotentoxin (**54**)	*A. porri*	[48]
	Dihydrotentoxin (**55**)	*A. citri*	[29]
		*A. porri*	[47,48]
	Uridine (**56**)	*A. alternata*	[49]
	Adenosine (**57**)	*A. alternata*	[49]
	Brassicicolin A (**58**)	*A. brassicicola*	[50,51]
	Fumitremorgin B (**59**)	*Alternaria* sp. FL25	[52]
	Fumitremorgin C (**60**)	*Alternaria* sp. FL25	[52]
	Paclitaxel = Taxol (**61**)	*A.* *alternata *var. *monosporus*	[53]
Steroids	Ergosterol (**62**)	*A. alternata*	[27,54]
	Ergosta-4,6,8(14),22-tetraen-3-one (**63**)	*A. alternata*	[27,54]
	Ergosta-4,6,8(9),22-tetraen-3-one (**64**)	*A.* *alternata*	[49]
	Ergosta-7,24(28)-dien-3-ol ( **65** )	*A. alternata*	[49]
	3β-Hydroxy-ergosta-5,8(9),22-trien-7-one (**66**)	*A. brassicicola* ML-P08	[55]
	3β,5α-Dihydroxy-ergosta-7,22-dien-6-one (**67**)	*A. brassicicola* ML-P08	[55]
	Cerevisterol (**68**)	*A. brassicicola* ML-P08	[55]
Terpenoids	Bicycloalternarene 1 (**69**)	*A. alternata*	[56]
	Bicycloalternarene 11 (**70**)	*A. alternata*	[56]
	Bicycloalternarene 2 (**71**)	*A. alternata*	[56]
	Bicycloalternarene 3 = ACTG toxin A (**72**)	*A. alternata*	[56]
	Bicycloalternarene 4 (**73**)	*A. alternata*	[56]
	Bicycloalternarene 10 (**74**)	*A. alternata*	[56]
	Bicycloalternarene 5 (**75**)	*A. alternata*	[56]
	Bicycloalternarene 8 (**76**)	*A. alternata*	[56]
	Bicycloalternarene 9 = ACTG toxin B (**77**)	*A. alternata*	[56]
	Bicycloalternarene 6 (**78**)	*A. alternata*	[56]
	Bicycloalternarene 7 (**79**)	*A. alternata*	[56]
	Tricycloalternarene 1a (**80**)	*A. alternata*	[57]
	Tricycloalternarene 1b (**81**)	*A. alternata*	[57,58]
	Tricycloalternarene 11a (**82**)	*A. alternata*	[59]
	Tricycloalternarene 11b (**83**)	*A. alternata*	[59]
	Tricycloalternarene 2a (**84**)	*A. alternata*	[57]
	Tricycloalternarene 2b (**85**)	*A. alternata*	[57,58]
	Tricycloalternarene 3a (**86**)	*A. alternata*	[57]
	Tricycloalternarene 3b = ACTG toxin G (**87**)	*A. alternata*	[57,60]
		*A. citri*	[61]
	ACTG toxin H (**88**)	*A. citri*	[61]
	Tricycloalternarenal (**89**)	*A. alternata*	[60]
	Tricycloalternarene 4a (**90**)	*A. alternata*	[57]
	Tricycloalternarene 4b (**91**)	*A. alternate*	[57]
	Tricycloalternarene 10b (**92**)	*A. alternate*	[59]
	Tricycloalternarene 5a (**93**)	*A. alternate*	[57]
	Tricycloalternarene 5b (**94**)	*A. alternate*	[57]
	Tricycloalternarene 8a (**95**)	*A. alternate*	[59]
	Tricycloalternarene 9b (**96**)	*A. alternate*	[59]
	Tricycloalternarene 6a (**97**)	*A. alternate*	[59]
	Tricycloalternarene 6b (**98**)	*A. alternate*	[59]
	Tricycloalternarene 7a (**99**)	*A. alternate*	[59]
	Tricycloalternarene 7b (**100**)	*A. alternate*	[59]
	Tricycloalternarene A (**101**)	*A.* *alternata* Ly83	[58]
	Tricycloalternarene B (**102**)	*A.* *alternata* Ly83	[58]
	Tricycloalternarene C (**103**)	*A. alternata* Ly83	[58]
	Tricycloalternarene D (**104**)	*A. alternata* Ly83	[58]
	Tricycloalternarene E (**105**)	*A. alternata* Ly83	[58]
	Brassicicene A (**106**)	*A. brassicicola*	[62]
	Brassicicene B (**107**)	*A. brassicicola*	[62]
	Brassicicene C (**108**)	*A. brassicicola*	[62]
	Brassicicene D (**109**)	*A. brassicicola*	[62]
	Brassicicene E (**110**)	*A. brassicicola*	[62]
	Brassicicene F (**111**)	*A. brassicicola*	[62]
	Brassicicene G (**112**)	*A. brassicicola*	[51]
	Brassicicene H (**113**)	*A. brassicicola*	[51]
	Brassicicene I (**114**)	*A. brassicicola*	[51]
	Abscisic acid = ABA (**115**)	*A. brassicae*	[63]
	(1aS,2S,6R,7R,7aR,7bR)-1a,2,4,5,6,7,7a,7b-Octahydro-7,7a-dimethyl-1a-(1-methylethenyl)-naphth[1,2-b]oxirene-2,6-diol (**116**)	*A. citri*	[61]
	Helvolic acid (**117**)	*Alternaria* sp. FL25	[43]
Pyranones	Radicinin (**118**)	*A. chrysanthemi*	[64,65]
		*A. helianthi*	[66]
		*A. radicina*	[67]
	Deoxyradicinin (**119**)	*Alternaria* sp. CIB 108	[68]
		*A. helianthi*	[66,69]
	Radicinol (**120**)	*A. chrysanthemi*	[64,65]
		*A. radicina*	[67]
	Deoxyradicinol (**121**)	*A. helia* *nthi*	[66]
	3-Epiradicinol (**122**)	*Alternaria* sp. CIB 108	[68]
		*A. chrysanthemi*	[65]
		*A. radicina*	[67]
	3-Epideoxyradicinol (**123**)	*Alternaria* sp. CIB 108	[68]
		*A. helianthi*	[70]
	3-Methoxy-3-epiradicinol (**124**)	*A. chrysanthemi*	[65]
	9,10-Epoxy-3-methoxy-3-epiradicinol (**125**)	*A. chrysanthemi*	[65]
	Radianthin (**126**)	*A. helianthi*	[66]
	3-Butyryl-6-[rel-(1S,2S)-1,2-dihydroxypropyl]-4-hydroxy-2H-pyran-2-one (**127**)	*Alternaria* sp. CIB 108	[68]
	Phomapyrone A = Phomenenin A (**128**)	*A. brassicicola*	[51]
		*A. infectoria*	[71]
	Phomenenin B (**129**)	*A. infectoria*	[71]
	Phomapyrone G (**130**)	*A. brassicicola*	[51]
	Infectopyrone (**131**)	*A.* *arbusti*	[72]
		*A. conju* *ncta*	[72]
		*A. infectoria*	[72,73]
		*A. intercepta*	[72]
		*A. metachromatica*	[72]
		*A. novae-zelandiae*	[72]
		*A. oregonensis*	[72]
		*A. triticimaculans*	[72]
		*A. viburni*	[72]
	Herbarin A (**132**)	*A. brassicicola* ML-P08	[55]
	Alternaric acid (**133**)	*A. solani*	[74]
	Novae-zelandin A (**134**)	*A. cetera*	[72]
		*A. infectoria*	[72]
		*A. intercepta*	[72]
		*A. novae-zelandiae*	[72]
		*A. triticimaculans*	[72]
		*A. viburni*	[72]
	Novae-zelandin B (**135**)	*A. cetera*	[72]
		*A. infectoria*	[72]
		*A. intercepta*	[72]
		*A. novae-zelandiae*	[72]
		*A. triticimaculans*	[72]
		*A. viburni*	[72]
	4 *Z*-Infectopyrone (**136**)	*A. arbusti*	[72]
		*A. conjuncta*	[72]
		*A. infectoria*	[72]
		*A. intercepta*	[72]
		*A. metachromatica*	[72]
		*A. novae-zelandiae*	[72]
		*A. oregonensis*	[72]
		*A. triticimaculans*	[72]
		*A. viburni*	[72]
	Pyrenocine A (**137**)	*A. infectoria*	[72]
	Pyrenocine B (**138**)	*A. infectoria*	[72]
	Pyrenocine C (**139**)	*A. infectoria*	[72]
	ACRL toxin I (**140**)	*A. citri*	[75]
	ACRL toxin II (**141**)	*A. citri*	[76]
	ACRL toxin III (**142**)	*A. citri*	[76]
	ACRL toxin IV (**143**)	*A. citri*	[76]
	ACRL toxin IV’ (**144**)	*A. citri*	[76]
	Solanapyrone A (**145**)	*A. solani*	[77]
	Solanapyrone B (**146**)	*A. solani*	[77]
	Solanapyrone C (**147**)	*A. solani*	[77]
	Solanapyrone D (**148**)	*A. solani*	[78]
	Solanapyrone E (**149**)	*A. solani*	[78]
	Tenuissimasatin (**150**)	*A. tenuissima*	[22]
	Altechromone A (**151**)	*A. brassicicola* ML-P08	[55]
	2,5-Dimethyl-7-hydroxychromone (**152**)	*Alternaria* sp.	[79]
	Phomapyrone F (**153**)	*A. brassicicola*	[51]
	Altenuisol (**154**)	*Alternaria* sp*.*	[80]
		*A. tenuis*	[81]
	Altertenuol (**155**)	*A. tenuis*	[82]
	Dehydroaltenusin (**156**)	*A. tenuis*	[83]
	Alternariol =AOH (**157**) Alternariol 5-*O*-sulfate (**158**)	*Alternaria* sp*.*	[41,84]
		*A. alternata*	[25,27,85]
		*Alternaria* sp.	[84]
	Alternariol 9-methyl ether = AME = Djalonensone (**159**)	*Alternaria* sp*.*	[41,84,86]
		*A. alternata*	[25,27,85]
		*A. linicola*	[31]
		*A. tenuis*	[87]
		*A. tenuissima*	[86]
	Alternariol 5-*O*-methyl ether-4'-*O*-sulfate (**160**)	*Alternaria* sp*.*	[84]
	3'-Hydroxyalternariol (**161**)	*Alternaria* sp*.*	[84]
	Altenuene = ATL (**162**)	*Alternaria* sp*.*	[84]
		*A. alternata*	[85]
	Isoaltenuene (**163**)	*A. alternata*	[88]
	4'-Epialtenuene (**164**)	*Alternaria* sp*.*	[84]
	5'-Epialtenuene (**165**)	*A. alternata*	[89]
	Neoaltenuene (**166**)	*A. alternata*	[89]
	Rubrofusarin B (**167**)	*A. alternata*	[23]
	Fonsecin (**168**)	*A. alternata*	[23]
	Fonsecin B (**169**)	*A. alternata*	[23]
	Aurasperone A (**170**)	*A. alternata*	[23]
	Aurasperone B (**171**)	*A. alternata*	[23]
	Aurasperone C (**172**)	*A. alternata*	[23]
	Aurasperone F (**173**)	*A. alternata*	[23]
Quinones	Macrosporin (**174**)	*Alternaria* sp. ZJ-2008003	[90]
		*A. porri*	[32]
		*A. solani*	[91]
	Demethylmacrosporin (**175**)	*A. porri*	[32]
	Dihydroaltersolanol A (**176**)	*Alternaria* sp. ZJ-2008003	[90]
	Tetrahydroaltersolanol B (**177**)	*Alternaria* sp. ZJ-2008003	[90]
		*A. solani*	[92]
	Tetrahydroaltersolanol C (**178**)	*Alternaria* sp. ZJ-2008003	[90]
	Tetrahydroaltersolanol D (**179**)	*Alternaria* sp. ZJ-2008003	[90]
	Tetrahydroaltersolanol E (**180**)	*Alternaria* sp. ZJ-2008003	[90]
	Tetrahydroaltersolanol F (**181**)	*Alternaria* sp. ZJ-2008003	[90]
	Bostrycin (**182**)	*A. eichhorniae*	[93]
	4-Deoxybostrycin (**183**)	*A. eichhorniae*	[93]
	Hydroxybostrycin (**184**)	*A. solani*	[94]
	Altersolanol A = Stemphylin (**185**)	*A. porri*	[95]
		*A. solani*	[94,96,97]
	Altersolanol B = Dactylarin (**186**)	*Alternaria* sp. ZJ-2008003	[90]
		*A. porri*	[95]
		*A. s* *olani*	[94,96,97]
	Altersolanol C = Dactylariol (**187**)	*Alternaria* sp. ZJ-2008003	[90]
		*A. porri*	[95,98]
		*A. solani*	[94,96,97]
	Altersolanol D (**188**)	*A. solani*	[94,96,97]
	Altersolanol E (**189**)	*A. solani*	[94,96,97]
	Altersolanol F (**190**)	*A. solani*	[94,96,97]
	Altersolanol G (**191**)	*A. solani*	[94]
	Altersolanol H (**192**)	*A. solani*	[94]
	Altersolanol L (**193**)	*Alternaria* sp. ZJ-2008003	[90]
	Ampelanol (**194**)	*Alternaria* sp. ZJ-2008003	[90]
	Alterporriol A/B (**195**)	*A. porri*	[32]
		*A. solani*	[94,99]
	Alterporriol C (**196**)	*Alternaria* sp. ZJ-2008003	[90]
		*A. porri*	[32]
		*A. solani*	[99]
	Alterporriol D/E (**197**)	*A. porri*	[32]
	Alterporriol F (**198**)	*A. porri*	[32]
	Alterporriol K (**199**)	*Alternaria* sp. ZJ9-6B	[100]
	Alterporriol L (**200**)	*Alternaria* sp. ZJ9-6B	[100]
	Alterporriol M (**201**)	*Alternaria* sp. ZJ9-6B	[100]
	Alterporriol N (**202**)	*Alternaria* sp. ZJ-2008003	[90]
	Alterporriol O (**203**)	*Alternaria* sp. ZJ-2008003	[90]
	Alterporriol P (**204**)	*Alternaria* sp. ZJ-2008003	[90]
	Alterporriol Q (**205**)	*Alternaria* sp. ZJ-2008003	[90]
	Alterporriol R (**206**)	*Alternaria* sp. ZJ-2008003	[90]
	Alterperylenol (**207**)	*Alternarial* sp.	[79,101]
		*Alternaria* sp. M6	[102]
		*A. alternata*	[27]
		*A. cassiae*	[103]
		*A. tenuissima*	[22]
	8β-Chloro-3,6aα,7β,9β,10-pentahydroxy-9,8,7,6a-tetrahydroperylen-4(6aH)-one (**208**)	*Alternaria* sp. M6	[102]
	Dihydroalterperylenol (**209**)	*Alternarial* sp.	[101]
		*Alternaria* sp. M6	[102]
		*A. alternate*	[104]
	Stemphyperylenol (**210**)	*Alternaria* sp.	[79]
		*A. alternata*	[105]
		*A. cassiae*	[103]
	6-Epi-stemphytriol (**211**)	*A. alternata*	[105]
	Altertoxin I = ATX-I (**212**)	*Alternaria* sp.	[79,80,106]
		*A. alternata*	[26,27,104,105,107]
		*A. cassiae*	[103]
		*A. tenuissima*	[22]
	Alteichin (**213**)	*A. alternata*	[26,107]
		*A. eichorniae*	[108]
	Alterlosin I (**214**)	*A. alternata*	[26]
	Alterlosin II (**215**)	*A. alternata*	[26]
Phenolics	*p*-Hydroxybenzoic acid (**219**)	*A. tagetica*	[109]
	Tyrosol (**220**)	*A. tagetica*	[109]
	α-Acetylorcinol (**221**)	*A. tenuissima*	[22]
	2-Carboxy-3-(2-hydroxypropanyl) phenol (**222**)	*Alternaria* sp. HS-3	[110]
	Methyl eugenol (**223**)	*Alternaria* sp.	[111]
	Tagetolone (**224**)	*A. tagetica*	[109]
	Tagetenolone (**225**)	*A. tagetica*	[109]
	Zinniol (**226**)	*A. carthami*	[112,113]
		*A. cichorii*	[33]
		*A. cirsinoxia*	[114]
		*A. dauci*	[115]
		*A. macrospora*	[113]
		*A. porri*	[113,116]
		*A. solani*	[113,117,118]
		*A. tagetica*	[113,116,119]
		*A. zinniae*	[120]
	8-Zinniol 2-(phenyl)-ethyl ether (**227**)	*A. solani*	[118]
		*A. tagetica*	[116]
	8-Zinniol methyl ether (**228**)	*A. solani*	[118]
		*A. tageti* *ca*	[116]
	8-Zinniol acetate (**229**)	*A. tagetica*	[116]
	7-Zinniol acetate (**230**)	*A. tagetica*	[116]
	Homozinniol (**231**)	*A. solani*	[117]
	Zinnol (**232**)	*A. cichorii*	[33]
	8-Zinnol methyl ether (**233**)	*A. solani*	[118]
		*A. tagetica*	[116]
	Zinnidiol (**234**)	*A. cichorii*	[33]
	2-(2'',3''-dimethyl-but-1-enyl)-Zinniol (**235**)	*A. solani*	[118]
	Bis-7-*O*-8''.8-*O*-7''-zinniol (**236**)	*A. tagetica*	[121]
	Bis-7-*O*-7''.8-*O*-8''-zinniol (**237**)	*A. tagetica*	[121]
	4-Acetyl-5-hydroxy-3,6,7-trimethylbenzofuran-2(3 *H*)-one (**238**)	*Alternaria* sp. HS-3	[110]
	5-Methyl-6-hydroxy-8-methoxy-3-methylisochroman (**239**)	*Alternaria* sp. HS-3	[110]
	Alternarian acid (**240**)	*Alternaria* sp.	[79]
	Altenusin (**241**)	*Alternaria* sp.	[79,84,122,123]
		*A. mali*	[124]
		*A. tenuis*	[82]
	Desmethylaltenusin (**242**)	*Alternaria* sp.	[84]
	Porric acid D (**243**)	*Alternaria* sp.	[123]
	Alterlactone (**244**)	*Alternaria* sp.	[84]
	Alternethanoxin A (**245**)	*A. sonchi*	[125]
	Alternethanoxin B (**246**)	*A. sonchi*	[125]
	Alternarienonic acid (**247**)	*Alternaria* sp.	[79,84]
	Talaroflavone (**248**)	*Alternaria* sp.	[84]
	Curvularin (**249**)	*A. cinerariae*	[126]
		*A. tomato*	[127]
	(4S)-α,β-Dehydrocurvularin (**250**)	*Alternaria* sp.	[86]
		*A. cinerariae*	[126,128]
		*A. tenuissima*	[86]
		*A. tomato*	[127]
		*A. zinniae*	[129]
	β-Hydroxycurvularin (**251**)	*A. tomato*	[127]
	Resveratrol (**252**)	*Alternaria* sp. MG1	[130]
	6-(3',3'-dimethylallyloxy)-4-Methoxy-5-methylphthalide (**253**)	*A. porri*	[7]
		*A. solani*	[117]
		*A. tagetica*	[116]
	Porritoxinol (**254**)	*A. porri*	[131]
	5-(3',3'-dimethylallyloxy)-7-Methoxy-6-methylphthalide (**255**)	*A. porri*	[7,32,34]
		*A. solani*	[118]
		*A. tagetica*	[116]
	Porriolide (**256**)	*A. porri*	[7,32]
Miscellaneous Metabolites	Depudecin (**257**)	*A. brassicicola*	[132]
	Altenin (**258**)	*A. kikuchiana*	[133]
	Brefeldin A (**259**)	*A. carthami*	[112]
		*A. zinniae*	[129]
	7-Dehydrobrefeldin A (**260**)	*A. carthami*	[112]
	α-Linoleic acid (**261**)	*A. infectoria*	[71]
	α-Linolenic acid (**262**)	*A. infectoria*	[71]
	AF-toxin I (**263**)	*A. alternata*	[134,135]
	AF-toxin II (**264**)	*A. alternata*	[134,135]
	AF-toxin III (**265**)	*A. alternata*	[134]
	Xanalteric acid I (**266**)	*Alternaria* sp.	[79]
	Xanalteric acid II (**267**)	*Alternaria* sp.	[79]
	Cladosporol (**268**)	*A. alternate* var. *monosporus*	[53]

### 2.1. Nitrogen-Containing Metabolites

The nitrogen-containing compounds such as amides, amines, and cyclopeptides have been isolated from *Alternaria* fungi. Some of them belong to the host-selective phytotoxins in host-parasite interactions [39].

#### 2.1.1. Amines and Amides

**Figure 1:**
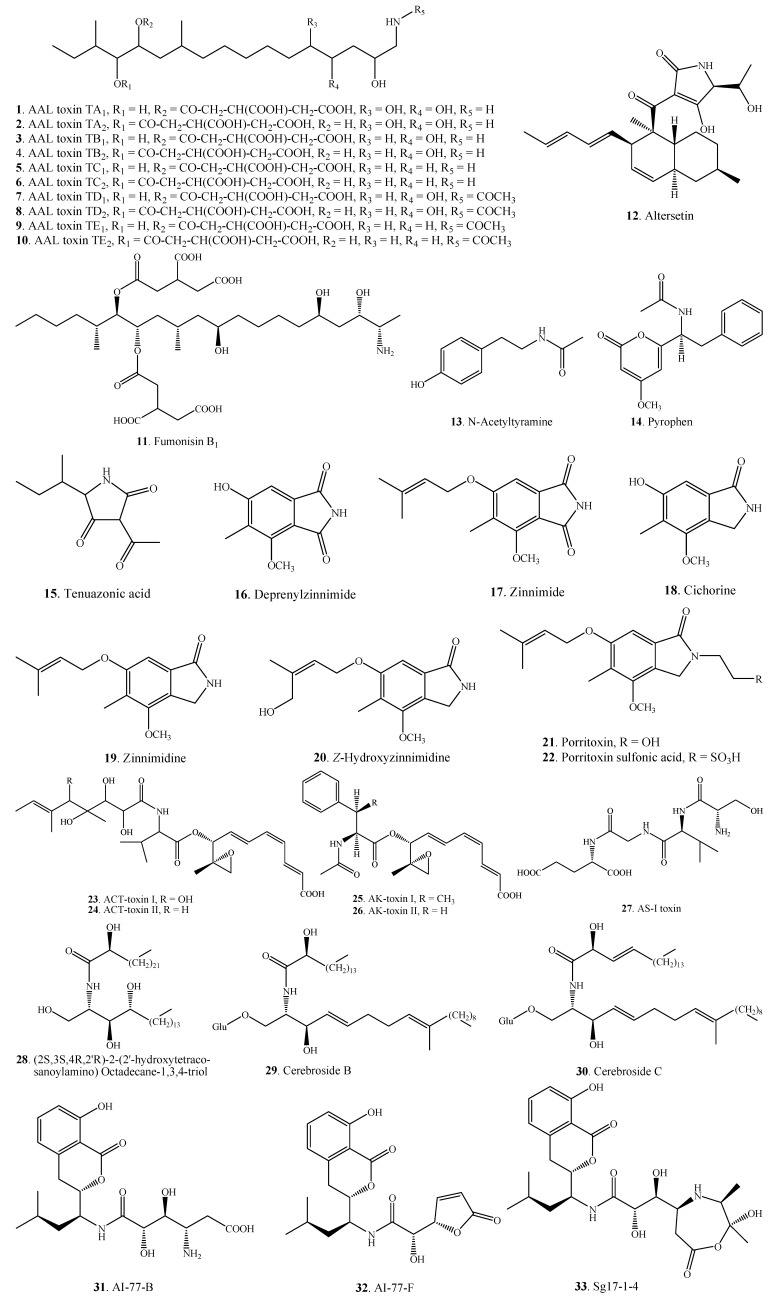
Amines and amides isolated from *Alternaria* fungi.

Amines and amides **1**–**33** are the common nitrogen-containing metabolites produced by *Alternaria* fungi (Figure 1). Ten sphinganine analogs designated AAL toxins **1**–**10** with an amino polyol backbone were isolated from *A. alternata* f.sp. *lycopersici* [16,17,18]. AAL toxins belong to host-specific phytotoxins. Very interestingly, AAL-toxins TB_1_ (**3**), TB_2_ (**4**), TC_1_ (**5**), TC_2_ (**6**), TD_1_ (**7**), TD_2_ (**8**), TE_1_ (**9**) and TE_2_ (**10**) have also been isolated from *Fusarium moniliforme* [136] and *F. verticillioides* [137]. Three amide alkaloids, AI-77-B (**31**), AI-77-F (**32**) and Sg17-1-4 (**33**), containing an isocoumarin structure were isolated from the marine fungus *Alternaria tenuis* Sg17-1 [42]. Other *Alternaria* amines and amides along with their distributions in *Alternaria* fungi are shown in Table 1.

#### 2.1.2. Cyclopeptides

**Figure 2:**
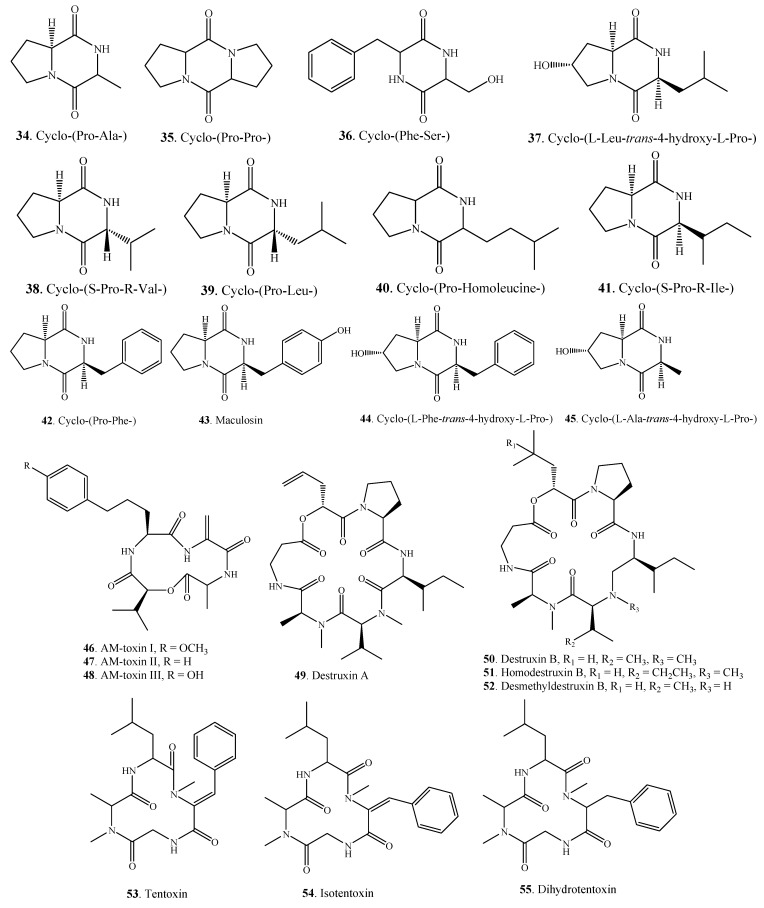
Cyclopeptides isolated from *Alternaria* fungi.

Some *Alternaria* fungi can produce cyclopeptides **34**–**55** which are shown in Figure 2. Seven cyclopeptides, namely cyclo-(Pro-Ala-) (**34**), cyclo-(Pro-Pro-) (**35**), cyclo-[l-Leu-*trans*-4-hydroxy-L-Pro-] (**37**), cyclo-(S-Pro-R-Val-) (**38**), cyclo-(Pro-Leu-) (**39**), cyclo-(S-Pro-R-Ile-) (**41**), and cyclo-(Pro-Phe-) (**42**) were isolated from the endophytic fungus *A. tenuissima* derived from the bark of *Erythrophleum fordii* Oliver (Leguminosae) [22].

Three diketopiperazine dipeptides, namely cyclo-[l-Leu-*trans*-4-hydroxy-L-Pro-] (**37**), cyclo-(l-Phe-*trans*-4-hydroxy-l-Pro-) (**44**), and cyclo-(l-Ala-*trans*-4-hydroxy-L-Pro-) (**45**) were extracted from culture broth of the grapevine endophyte *A. alternata* [44].

Two cyclopeptides destruxins A (**49**) and B (**50**) were isolated from *A. linicola* [31]. Destruxin B (**50**) was also found in *A. brassicae* as the major phytotoxin [45]. Other cyclopeptides along with their distributions in *Alternaria* fungi are shown in Table 1.

#### 2.1.3. Other Nitrogen-Containing Metabolites

**Figure 3:**
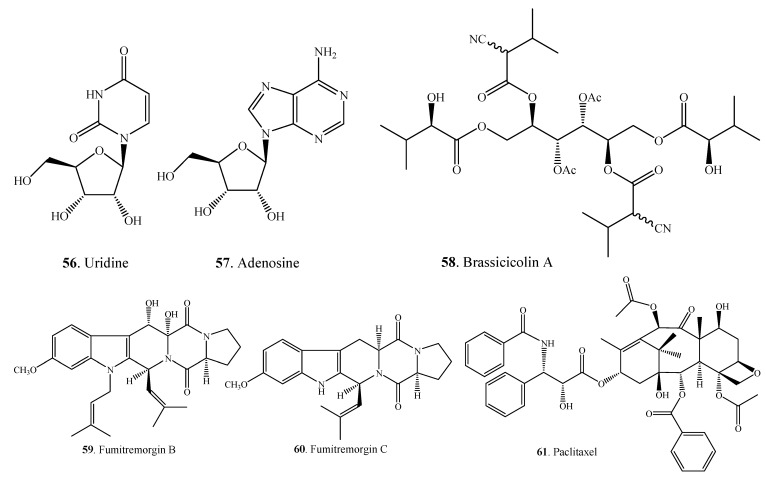
Other nitrogen-containing metabolites isolated from *Alternaria* fungi.

Other nitrogen-containing metabolites isolated from *Alternaria* fungi are shown in Figure 3. Two nucleosides namely uridine (**56**) and adenosine (**57**) were isolated from *A. alternata* [49].

Brassicicolin A (**58**), an isocyanide metabolite, was isolated as a mixture of epimers from *A. brassicicola* which was the pathogen of *Brassica* species [50,51]. Two indole alkaloids fumitremorgins B (**59**) and C (**60**) were produced by the endophytic fungus *Alternaria* sp. FL25 from *Ficus carica* (Moraceae) [52]. Paclitaxel (taxol, **61**), a diterpenoid alkaloid with antitumor activity, was isolated from the endophytic fungus *A. alternata* var. *monosporus* obtained from the inner bark of *Taxus yunnanensis* (Taxaceae) [53]. Paclitaxel has also been isolated from yew trees (*Taxus* spp.) and their cell cultures [140,142].

### 2.2. Steroids

**Figure 4:**
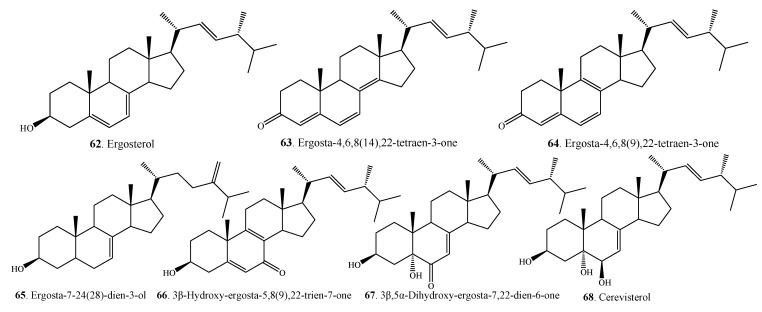
Steroids isolated from *Alternaria* fungi.

Some steroids (**62**–**68**) have been isolated from *Alternaria* fungi (Figure 4 and Table 1). These findings are consistent with the considerations that ergosterol (**62**) and their derivatives are common to all fungi and occur widely among the fungi [143].

### 2.3. Terpenoids

**Figure 5:**
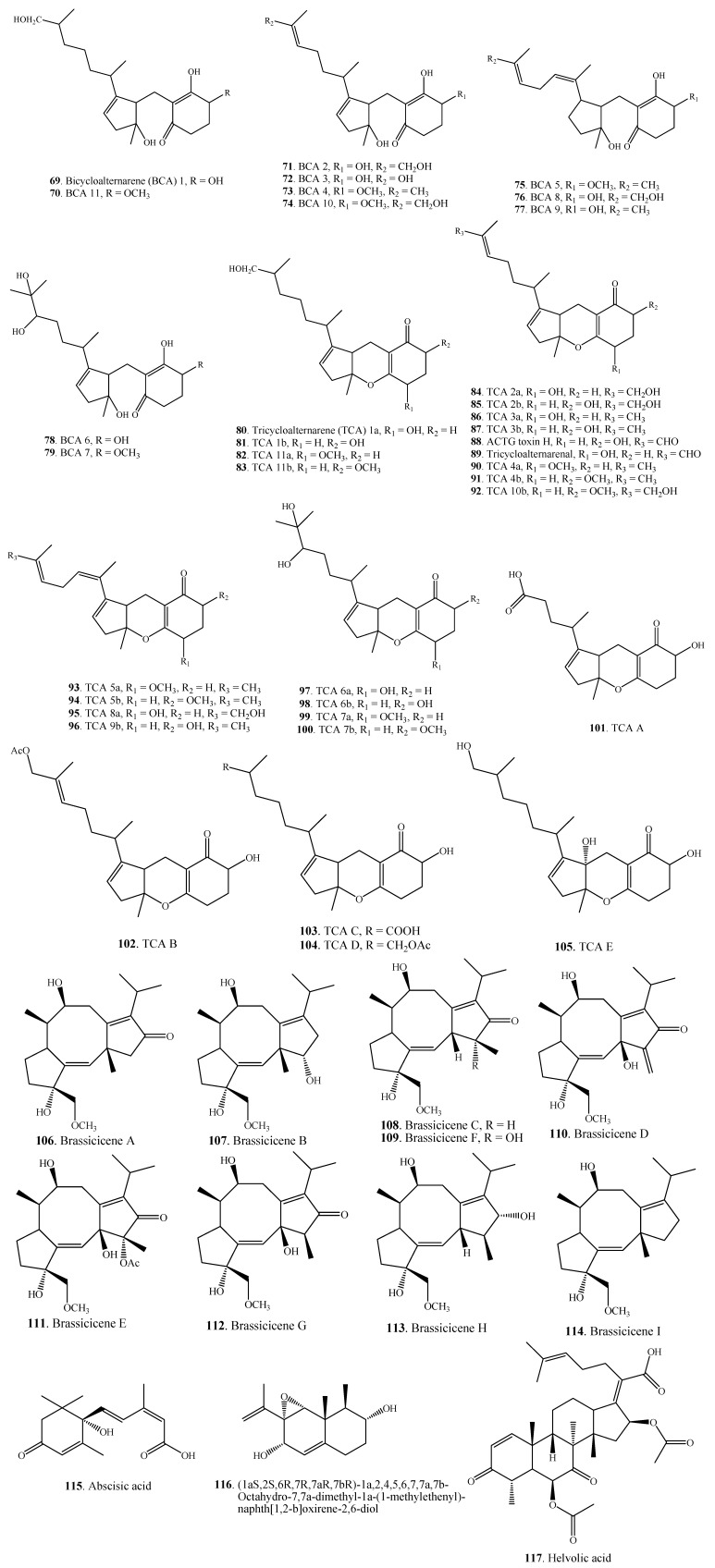
Terpenoids isolated from *Alternaria* fungi.

Most of terpenoids from *Alternaria* fungi have been found as the mixed terpenoids which have a multiple biogenesis (**69**–**105**). Other *Alternaria* terpenoids include diterpenoids **106**–**114**, sesquiterpenoids **115**,**116** and a triterpenoid **117**, which are shown in Figure 5.

Eleven bicycloalternarenes (BCAs, **69**–**79**) were isolated and characterized from the culture filtrate of the phytopathogenic fungus *A. alternata* [56].

Nineteen tricycloalternarenes (TCAs) were isolated from the culture filtrate of the phytopathogenic fungus *A. alternata* from *Brassica sinensis* (Cruciferae). Tricycloalternarenes are closely related to ACTG toxins **87**,**88**. Structural differences mainly occur in the isoprenoid side chain and the substitution pattern of the C-ring of the tricycloalternarenes [57,58,59,60].

Two tricycloalternarenes, ACTG toxins G (TCA 3b, **87**) and H (**88**), along with a sesquiterpene (1aS,2S,6R,7R,7aR,7bR)-1a,2,4,5,6,7,7a,7b-octahydro-7,7a-dimethyl-1a-(1-methylethenyl)-naphth [1,2-b] oxirene-2,6-diol (**116**) were isolated from culture broth of *A. citri*, the pathogen causing brown spot disease of mandarin (*Citrus reticulata*) [61].

Nine fusicoccane diterpenes designated brassicicenes A-I **106**–**114** were isolated from the culture filtrate of the canola pathogen *A. brassicicola* [51,52,53,54,55,56,57,58,59,60,61,62].

Abscisic acid (ABA, **115**), a sesquiterpenoid with plant growth regulation activity, was isolated from *A. brassicae*, a black spot pathogen of *Brassica* species (Cruciferae) [63].

Helvolic acid (**117**), a nortriterpenoid, was isolated from *Alternaria* sp. FL25, an endophytic fungus from *Ficus carica* (Moraceae) [43]. This metabolite (**117**) has also been isolated from *Aspergillus fumigatus* [138] and *Pichia guilliermondii* [139].

### 2.4. Pyranones

Pyranones are also called pyrones which include α-, β- and γ-pyranones. Most of the pyranones isolated from *Alternaria* fungi belong to α-pyranones.

#### 2.4.1. Simple Pyranones

**Figure 6:**
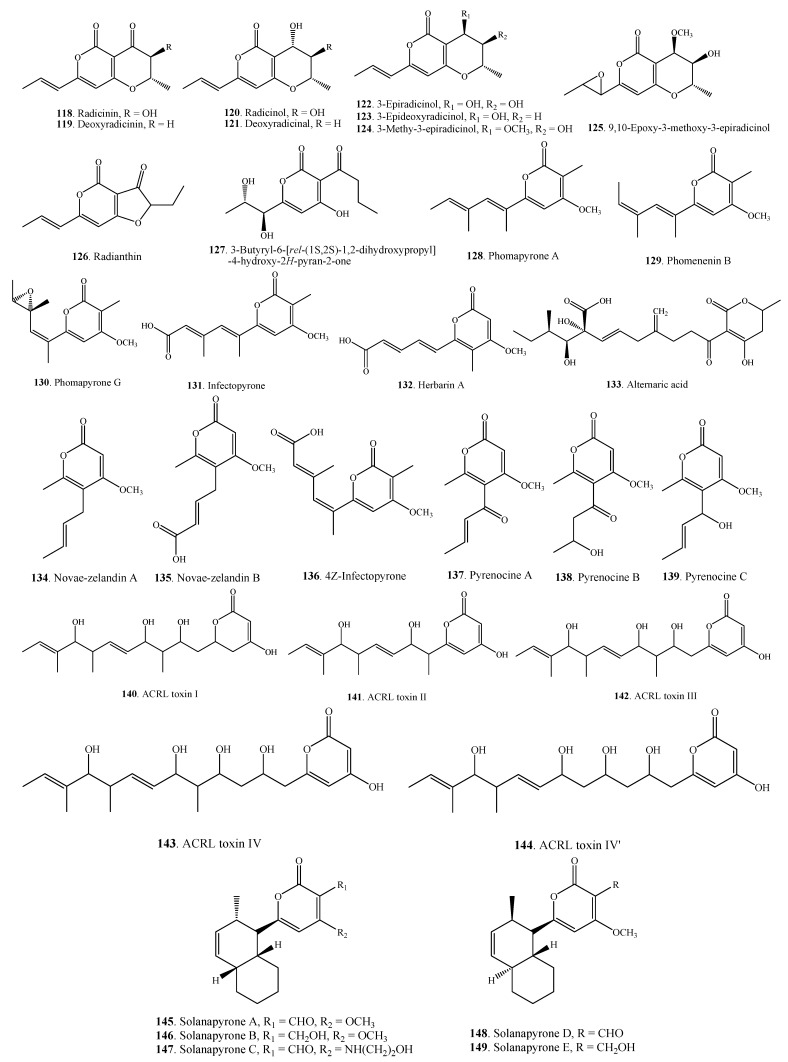
Simple pyranones isolated from *Alternaria* fungi.

The pyranones that do not contain benzene ring structure are defined as simple pyranones which belong to polyketides. Simple pyranones **118**–**149** from *Alternaria* fungi are shown in Figure 6. Three phytotoxins, ACRL toxins I (**140**), II (**141**) and III (**142**), with an α-dihydropyrone ring were isolated from *A. citri*, the causal agent of lemon (*Citrus limon*) [75,76].

Four metabolites namely novae-zelandins A (**134**) and B (**135**), 4*Z*-infectopyone (**136**), and infectopyrone (**131**) isolated from *A. infectoria* were thought to be important chemotaxonomic markers in the species group of *A. infectoria* [72].

#### 2.4.2. Monobenzopyranones

**Figure 7:**
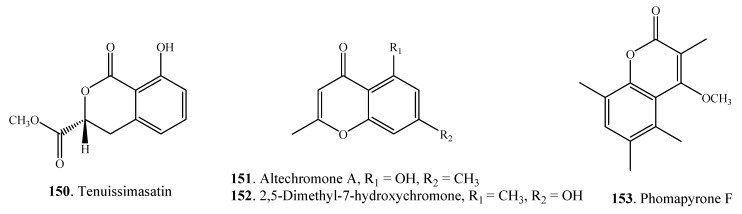
Monobenzopyranones isolated from *Alternaria* fungi.

Both benzo-α-pyranones and benzo-γ-pyranones have been found in *Alternaria* species (Figure 7 and Table 1). Benzo-α-pyranones are also called coumarin or isocoumarin derivatives. Four monobenzopyranones namely tenuissimassatin (**150**), altechromone A (**151**), 2,5-dimethyl-7-hydroxychromone (**152**) and phomapyrone F (**153**) were isolated from *Alternaria* fungi [22,55,79].

#### 2.4.3. Dibenzopyranones

**Figure 8:**
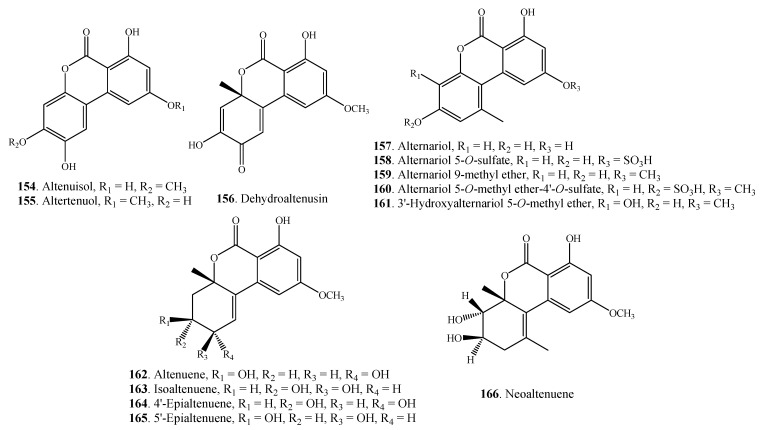
Dibenzopyranones isolated from *Alternaria* fungi.

A few dibenzo-α-pyranones **154**–**166** have been found in *Alternaria* fungi so far. They are shown in Figure 8. Both alternariol (AOH, **157**) and alternariol 9-methyl ether (AME, **159**) represent the main toxic metabolites of *Alternaria* fungi.

#### 2.4.4. Naphthopyranones

**Figure 9:**
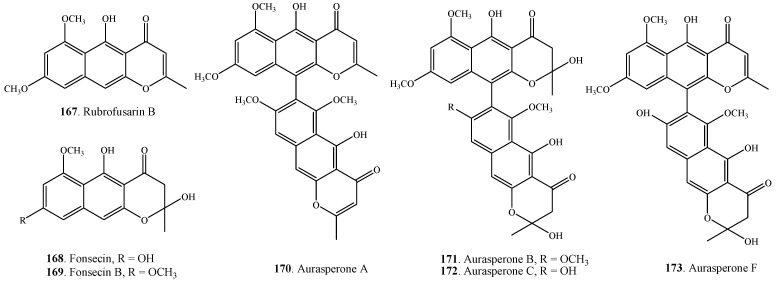
Naphthopyranones isolated from *Alternaria* fungi.

Seven naphtha-γ-pyranones **167**–**173** were found in *A. alternata* isolated from the marine soft coral *Denderonephthya hemprichi* (Figure 9). Among them, aurasperones A (**170**), B (**171**), C (**172**) and F (**173**) were dimeric naphtha-γ-pyranones [23].

### 2.5. Quinones

Two groups of quinones, anthraquinone and perylenequine derivatives have been isolated in *Alternaria* fungi so far.

#### 2.5.1. Anthraquinones

**Figure 10:**
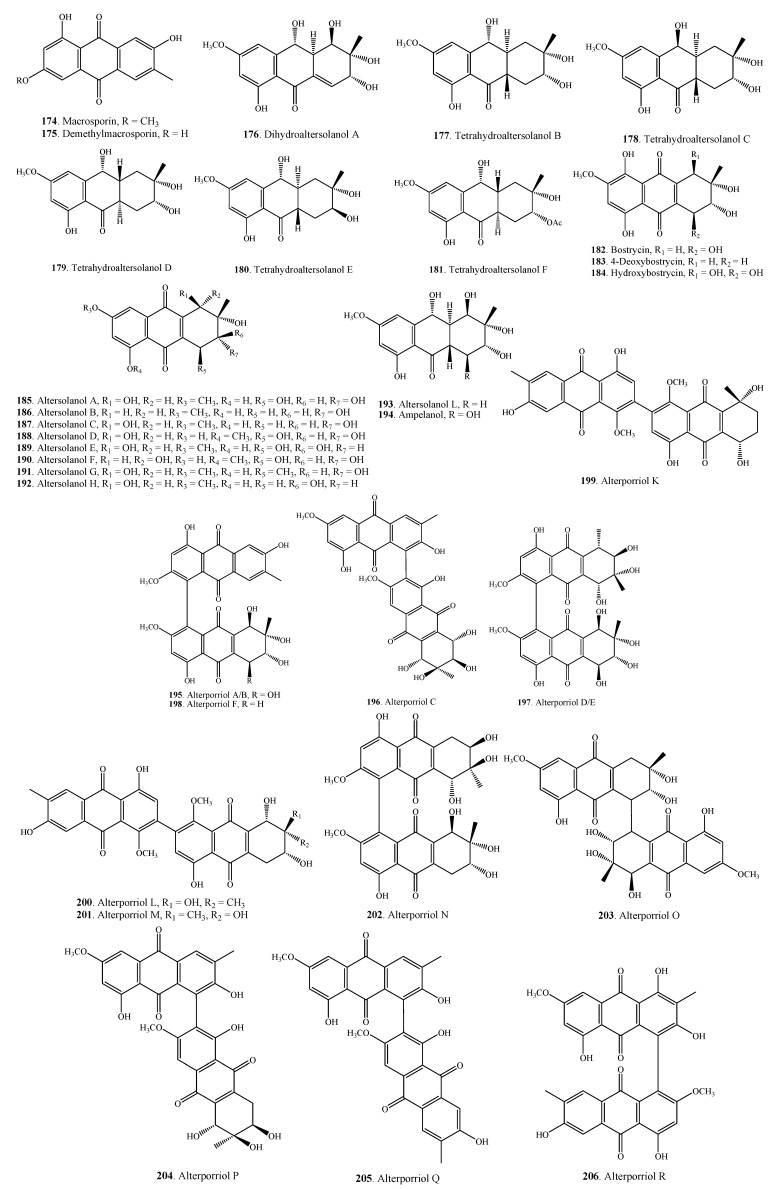
Anthraquinones isolated from *Alternaria* fungi.

Figure 10 shows the structures of twenty-one simple anthraquinones **174**–**194** and twelve bianthraquinones **195**–**206** from *Alternaria* fungi. Nine tetrahydroanthraquinones **174**–**183**, hydroxybostrycin (**184**) along with altersolanols A (**185**), B (**186**), C (**187**), D (**188**), E (**189**), F (**190**), G (**191**) and H (**192**) were isolated from *A. solani*, a causal pathogen of black spot disease on tomato (*Lycopersicon esculentum*) leaves [94,96].

Four bianthraquinones, alterporiols A/B (**195**), C (**196**), D/E (**197**), and F (**198**) were isolated from *A. porri*, the critical pathogen associated with the purple blotch disease of onion (*Allium cepa*) [32]. Three other bianthraquinones, alterporriols K (**199**), L (**200**) and M (**201**) were obtained from the mangrove endophytic fungus *Alternaria* sp. ZJ9-6B [100].

#### 2.5.2. Perylenequinones

**Figure 11:**
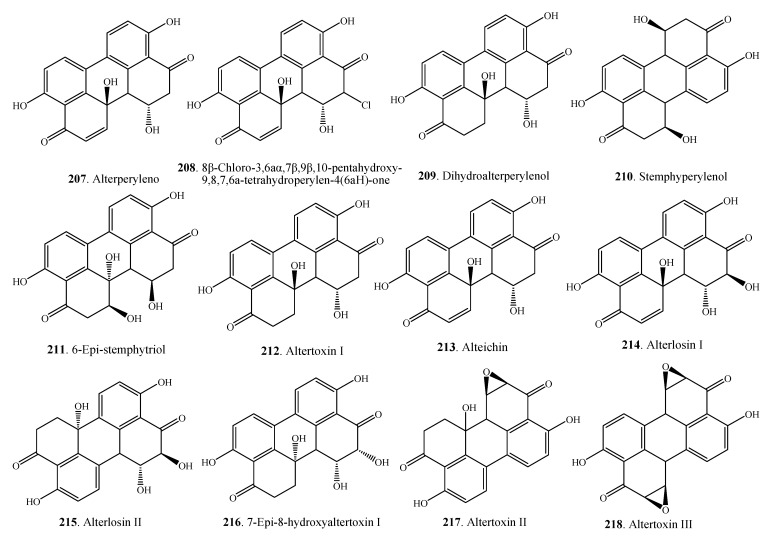
Perylenequinone derivatives isolated from *Alternaria* fungi.

The perylenequinones are a class of metabolites characterized by a pentacyclic conjugated chromophore. *Alternaria* fungi produce a variety of partially reduced perylenequinone derivatives. A monochloridated perylenequinone namely 8β-chloro-3,6aα,7β,9β,10-pentahydroxy-9,8,7,6a-tetrahydroperylen-4(6aH)-one (**208**) along with alterperylenol (**207**) and dihydroalterperylenol (**209**) were isolated from a halotolerant fungus *Alternaria* sp. M6 obtained from the solar salt field at the beach of Bohai Bay in China [102]. Other perylenequinones **207**–**218** are shown in Figure 11.

### 2.6. Phenolics

**Figure 12:**
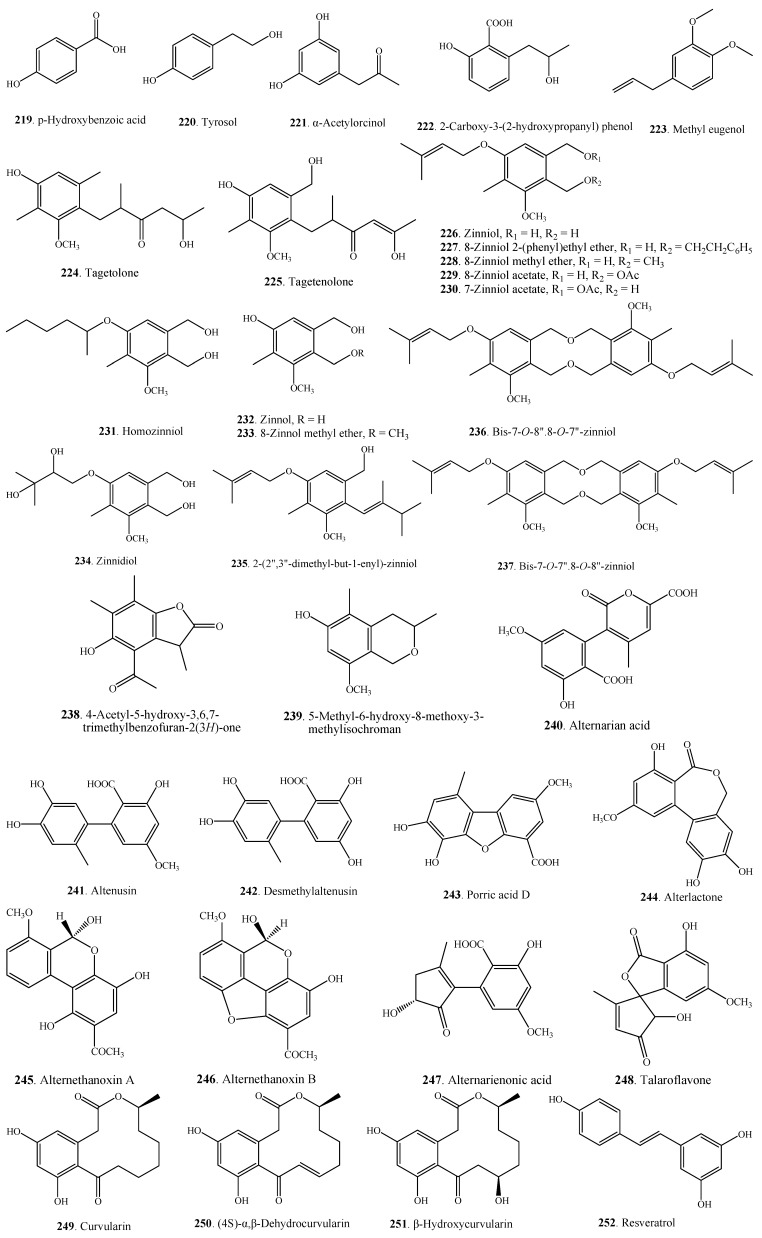
Phenolic metabolites isolated from *Alternaria* fungi.

**Figure 13:**
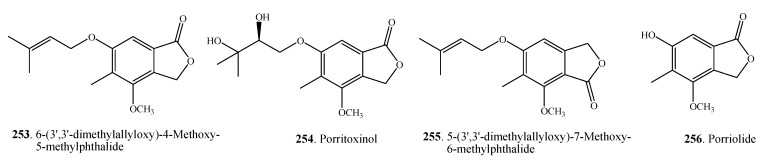
Phthalides isolated from *Alternaria* fungi.

The phenolic metabolites **219**–**256** from *Alternaria* fungi are shown in Figure 12, Figure 13. Most of them have a polyketide origin. One phenylpropanoid component was identified as methyl eugenol (**223**) by GC-MS from the volatile oil obtained by hydrodistillation from the *Alternaria* species isolated as the endophyte of rose (*Rosa damascaena*) [111]. Methyl eugenol (**223**) has been used as a flavouring agent in jellies, baked goods, non-alcoholic beverages, chewing gum, candy, pudding, relish, and ice cream [144].

Zinniol (**226**) along with its two analogues, bis-7-*O*-8''.8-*O*-7''-zinniol (**237**) and bis-7-*O*-7''.8-*O*-8''-zinniol (**238**), were isolated from the culture filtrate of *A. tagetica*, which was the causal agent of early blight in marigold (*Tagetes erecta*) [121].

One *Alternaria* species MG1 as the endophytic fungus from *Vitis vinifera* L. cv. Merlot could produce resveratrol (3,5,4'-trihydroxystilbene, **252**) [130]. Resveratrol has been known for preventing and slowing the occurrence of some human diseases, including cancer, cardiovascular disease, and ischemic injuries. It has also been shown that resveratrol (**252**) can enhance stress resistance and extend the lifespan of various organisms ranging from yeasts to vertebrates [145]. Resveratrol has been found in a variety of plant species such as *Vitis vinifera*, *Polygonum cuspidatum*, and *Glycine max* [141]. Endophytic *Alternaria* species for producing plant-derived resveratrol should be an important and novel resource with its potential application in pharmaceutical industry [146].

Phthalides are considered as a special group of phenolic compounds. Four phthalates **253**–**256** were isolated from *Alternaria* fungi that are shown in Figure 13, and their occurrences are shown in Table 1.

### 2.7. Miscellaneous Metabolites

**Figure 14:**
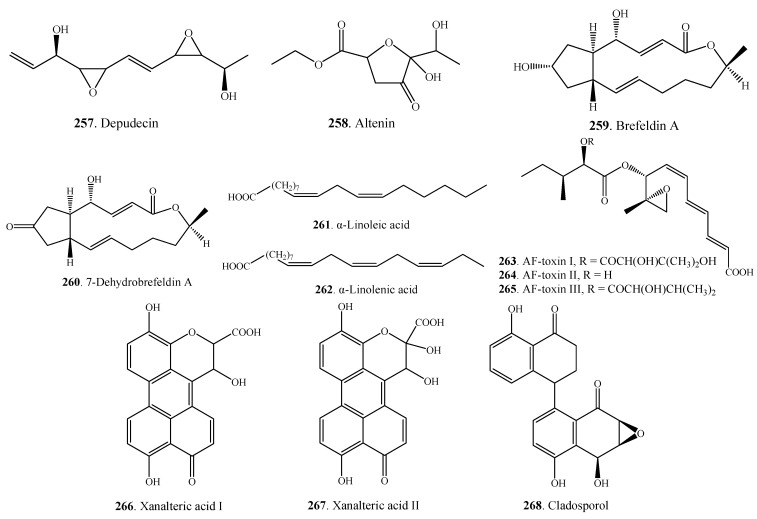
Miscellaneous metabolites isolated from *Alternaria* fungi.

The miscellaneous metabolites **257**–**268** isolated from *Alternaria* fungi are shown in Figure 14. Depudecin (**257**) was an eleven-carbon linear polyketide isolated from *A. brassicicola* [132]. Two carboxylic acids namely xanalteric acids I (**266**) and II (**267**) were isolated from the endophytic fungus *Alternaria* sp. from the mangrove plant *Sonneratia alba* (Sonneratiaceae) [79].

## 3. Biological Activities and Functions

*Alternaria* metabolites with diverse chemical properties have been clarified (Figure 1, Figure 2, Figure 3, Figure 4, Figure 5, Figure 6, Figure 7, Figure 8, Figure 9, Figure 10, Figure 11, Figure 12, Figure 13, Figure 14, Table 1). Some of them act as phytotoxins to plants or as mycototoxins to humans and animals. They have been examined to have a variety of biological activities and functions, which mainly include the effects on plants, cytotoxic and antimicrobial activities.

### 3.1. Effects on Plants

Plant pathogenic *Alternaria* species can affect cereals, vegetables and fruit crops in the field and during storage. *Alternaria* fungi contamination is responsible for some of the world’s most devastating plant diseases, causing serious reduction of crop yields and considerable economic losses. The metabolites from plant pathogenic fungi are usually toxic to plants and are called phytotoxins. They were further divided into host-specific and host non-specific toxins. The host-specific toxins (HSTs) are toxic only to host plants of the fungus that produces the toxin [6,13]. Another definition seems to be more acceptable that the host-specific toxins are toxic to plants that host the pathogen, but have lower phytotoxicity on non-host plants [147,148]. Most HSTs are considered to be pathogenicity factors, which the fungi producing them require to invade tissue and induce disease [149] All isolates of the pathogen that produce an HST are pathogenic to the specific host. All isolates that fail to produce HSTs lose pathogenicity to the host plants. Plants that are susceptible to the pathogen are sensitive to the toxin. Such correlations between HST production and pathogenicity in the pathogens, and between toxin sensitivity and disease susceptibility in plants provide persuasive evidence that HSTs can be responsible for host-specific infection and disease development. Johnson and coworkers revealed that the genes involved in HST synthesis such as the cyclopeptide synthetase gene, whose product catalyzed AM toxin production in *A. alternata* apple pathotype, might reside on a conditionally dispensable (CD) chromosome. The loss of the CD chromosome led to loss of both toxin production and pathogenicity without affecting fungal growth [150]. On the other hand, the exact roles of non-specific toxins in pathogenesis are largely unknown, but some are thought to contribute to the features of virulence, such as the symptom development and *in planta* pathogen propagation [6]. The virulence and host-specificity of these pathogens are based on production of the distinctive HSTs [13]. For *Alternaria* pathogens, there are now at least nine diseases caused by *Alternaria* species in which HSTs are responsible for fungal pathogenicity (Table 2). Most of *Alternaria* HSTs are nitrogen-containing metabolites.

**Table 2:** Host-specific phytotoxins from *Alternaria* fungi.

Phytotoxin name	*Alternaria* species	Host plant	Plant disease	Reference
AAL-toxins TA_1_ (**1**), TA_2_ (**2**), TB_1_ (**3**), TB_2_ (**4**), TC_1_ (**5**), TC_2_ (**6**), TD_1_ (**7**), TD_2_ (**8**), TE_1_ (**9**), TE_2_ (**10**)	*A. alternata* f.sp*. lycopersici*	Tomato (*Solanum lycopersicum*)	Stem canker disease of tomato	[16,17,18]
ACT-toxins I (**23**) and II (**24**)	*A. citri* (*A. alternata*)	Mandarins and tangerine (*Citrus* spp.)	Brown spot of tangerine	[37,38]
AK-toxins I (**25**) and II (**26**)	*A. kikuchiana* (*A. alternata*)	Japanese pear (*Pyrus serotina*)	Black spot disease	[39,135]
AS-I toxin (**27**)	*A. alternata*	Sunflower (*Helianthus annuus*)	Necrotic spots on sunflower leaves	[40]
Maculosin (**43**)	*A. alternata*	Spotted knapweed (*Centaurea maculosa*)	Black leaf blight	[10,26]
AM-toxins I (**46**), II (**47**) and III (**48**)	*A.mali* (*A. alternata*)	Apple (*Malus pumila*)	*Alternaria* blotch of apple	[39]
Destruxin A (**49**), Destruxin B (**50**), Homodestruxin B (**51**), Desmethyldestruxin B (**52**)	*A. brassicae*	*Brassica juncea*; *Brassica napus*; *Brassica rapa*	*Alternaria* blackspot disease of *Brassica*	[46,148]
ACRL toxins I (**140**), II (**141**), III (**142**), IV (**143**), IV’(**144**)	*A. citri*	Rough lemon (*Citrus limon*)	Brown spot disease of *Citrus*	[75,76]
AF-toxins I (**263**), II (**264**) and III (**265**)	*A. alternata*	Strawberry (*Fragaria* spp.)	*Alternaria* balck spot of strawberry	[134,135]

Among the HSTs, AAL toxins from tomato stem canker pathogen (*A. alternata* f.sp. *lycopercici*) have received a special attention. They were toxic to all tissues of sensitive tomato cultivars at low concentrations and induced apoptosis in sensitive tomato plants [151], and were found to inhibit *de novo* sphingolipid (ceramide) biosynthesis *in vitro*. Therefore, AAL toxins are called sphinganine-analog mycotoxins (SAMs). It has been reported that the tomato *Alternaria* stem canker locus mediated resistance to SAMs-induced apoptosis [152].

Destruxins are another group of HSTs produced both *in vitro* and *in planta* by *A. brassicae*, the causal agent of *Alternaria* blackspot disease of rapeseed and canola [148]. These cyclodepsipeptides exhibited a wide variety of biological activities such as antitumor, antiviral, insecticidal, cytotoxic, immunosuppressant, and antiproliferative effects except their phytotoxicity [153].

**Figure 15:**
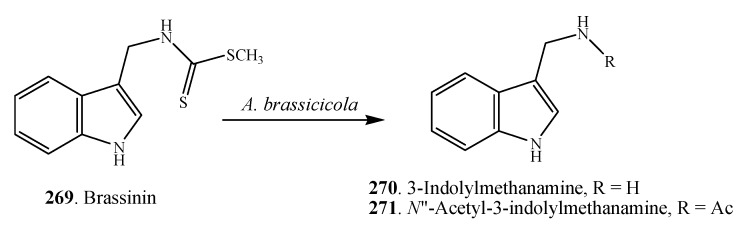
Detoxification pathway of the phytoalexin brassinin (**269**) by the pathogen *A. brassicicola* [51].

**Figure 16:**
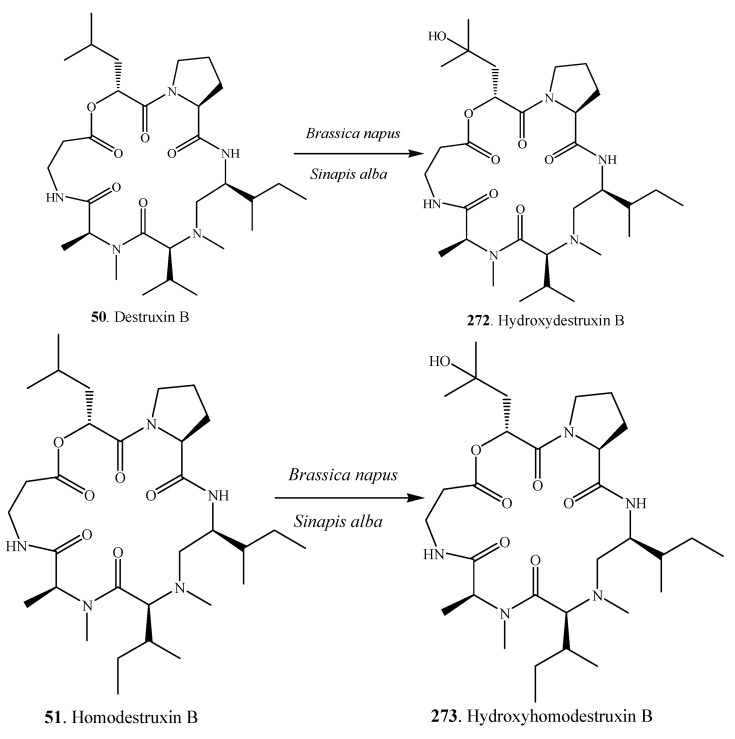
Detoxification pathway of the phytotoxins destruxin B (**50**) and homodestruxin B (**51**) by the hosts *Brassica napus* and *Sinapis alba* [155,156].

Interactions between *Alternaria* species and cruciferous plants were studied in detail by the Pedras group [51]. Nectrophic phytopathogens such as *A. alternata* and *A. brassicae* are known to synthesize phytotoxins that damage plant tissues and facilitate colonization, while in response to pathogen attack crucifers biosynthesize phytoanticipins and phytoalexins. Phytoalexins are secondary metabolites produced de novo by plants in response to diverse forms of stress including microbial infection, UV irradiation, and heavy metal salts, whereas phytoanticipins are constitutive defenses whose concentrations can increase upon stress [154]. To the detriment of cruciferous plants, the phytopathogens can overcome phytoanticipins and phytoalexins by producing detoxifying enzymes. For example, the phytoalexin brassinin (**269**) was detoxified into 3-indolylmethanamine (**270**) and *N*''-acetyl-3-indolylmethanamine (**271**) by the pathogen *A. brassicae* (Figure 15) [51]. Very interestingly, cruciferous plants (*i.e*., *Brasicca napus* and *Sinapis alba*) can convert host-specific toxins destruxin B (**50**) and homodestruxin B (**51**) into less phytotoxic hydrodestruxin B (**272**) and hydroxyhomodestruxin B (**273**), respectively (Figure 16) [155,156].

Host non-specific *Alternaria* phytotoxins can affect many plants regardless of whether they are a host or non-host of the pathogen [6,13]. Host non-specific nitrogen-containing phytotoxins include tenuazonic acid (**15**), porritoxin (**21**) and tentoxin (**53**). Tentoxin (**53**), a cyclic tetrapeptide from *A. alternata*, inhibited chloroplast development, which phenotypically manifests itself as chlorotic tissue [157]. Tentoxin (**53**) was suggested to exert its effect on chlorophyll accumulation through overenergization of thylakoids [158]. Tenuazonic acid (TeA, **15**) was investigated in *Chlamydomonas reinhardtii* thylakoids which revealed that TeA inhibited photophosphorylation with the action site at Q_B_ level [159].

Host non-specific pyranone phytotoxins include radicinin (**118**), deoxyradicinin (**119**), alternaric acid (**133**), alternuisol (**154**), altertenuol (**155**), dehydroaltenusin (**156**), alternariol (AOH, **157**), alternariol 9-methyl ether (AME, **159**), and alternuene (**162**). They are very common non-specific phytotoxic metabolites of *Alternaria* species [64,65,66,67,68,69,74,80,81,82,83,84,85].

Host non-specific quinone phytotoxins included bostrycin (**182**), 4-dexoybostrycin (**183**), and altersolanols A (**185**), B (**186**) and C (**187**) [93,94,95]. Altersolanol A (**185**), a tetrahydroanthraquinone phytotoxin from the culture broth of *A. solani*, inhibited the growth of cultured cells of *Nicotiana rustica*. It acted as a potent stimulator of NANH oxidation in the mitochondria isolated from *N. rustica* cells. Altersolanols acted as electron acceptors in an enzyme preparation of diaphorase. The capacity of altersolanols A, B, C, D, E and F to act as electron acceptors was in the order of A > E > C > B > F > D [160].

Host non-specific phenolic phytotoxins include zinniol (**226**) and its analogues **227**–**237**. Zinniol (**226**) from the liquid cultures of *A. tagetica* induced leaf tissue necrosis in a number of unrelated plant species (*Avena sativa*, *Cucumis sativus*, *Daucu carota*, *Hordeum vulgare*, *Triticum aestivum*) from different families which demonstrated that zinniol acted as a non host-specific phytotoxin [161]. However, Qui *et al.* evaluated the effects of zinniol at the cellular level and showed that pure zinniol was not obviously phytotoxic at concentrations known to induce necrosis in leaves of *Tagetes erecta*, which indicated that the classification of zinniol as a host non-specific phytotoxin should be further investigated [162].

Other host non-specific phytotoxins include α,β-dehydrocurvularin (**250**) and brefeldin A (**259**) from *A. zinniae*. They showed phytotoxic activity on *Xanthium occidentale*, a widespread noxious weed of Australian summer crops and pastures. The fungus *A. zinniae* and its toxins may be used as the mycoherbicides in integrated weed management programs [129].

Some fungal phytotoxins were toxic to weed species to show their herbicidal potentials in agriculture and forestry [10,163,164,165]. Some examples are shown in Table 3. Weed pathogens should be a very promising source of bioactive natural products for weed control. Tentoxin (**53**) was transformed to isotentoxin (**54**) by UV irradiation. Isotentoxin (**54**) had stronger wilting effects than tentoxin against the weed *Galium aparine* [11].

**Table 3:** Some examples of *Alternaria* phytotoxins which are toxic to weed species.

Phytotoxin name	*Alternaria* species	Target weed species	Reference
AAL-toxins ( **1**–**10**)	*A. alternata*	Jimson weed ( *Datura stramonium*)	[166]
Tenuazonic acid ( **15**)	*A. alternata*	*Lantana camara*	[12]
Maculosin ( **43**)	*A. alternata*	Spotted knapweed ( *Centaurea maculosa*)	[10]
Tentoxin ( **53**)	*A. alternata*	*Galium aparine*	[11]
Isotentoxin ( **54**)	*A. alternata*	*Galium aparine*	[11]
Alteichin ( **213**)	*A. eichorniae*	Water hyacinth ( *Eichhornia crassipes*)	[108]
Alternethanoxin A ( **245**)	*A. sonchi*	*Sonchus arvensis*	[125]
Alternethanoxin B ( **246**)	*A.* *sonchi*	*Sonchus arvensis*	[125]
Brefeldin A ( **259**)	*A. zinniae*	*Xanthium occidentale*	[129]

### 3.2. Cytotoxic Activity

Some *Alternaria* metabolites have been screened to show cytotoxic activity. They were thought as the potential sources for possible cancer chemopreventive agents. Porritoxin (**21**) was examined to have anti-tumor-promoting activity [7]. Three amides, AI-77-B (**31**), AI-77-F (**32**) and Sg17-1-4 (**33**), from a marine fungus *A. tenuis* Sg17-1 exhibited cytotoxic activity. Al-77-B (**31**) exhibited the cytotoxic activity on human malignant A375-S2 and human cervical cancer Hela cells with IC_50_ values of 0.1 and 0.02 mM, respectively. AI-77-F (**32**) showed a weak activity to Hela cells with an IC_50_ value of 0.4 mM. Sg17-1-4 (**33**) showed moderate activity with IC_50_ values of 0.3 and 0.05 mM, on malignant A375-S2 and Hela cells, respectively [42].

Of *Alternaria* dibenzopyranones, alternariol (**157**) was the most active metabolite to have cytotoxic activity on L5178Y mouse lymphoma cells [84], as well as to have inhibitory activity on protein kinase and xanthine oxidase [55]. Further investigation showed that alternariol (**157**) has been identified as a topoisomerase I and II poison which might contribute to the impairment of DNA integrity in human colon carcinoma cells [167]. It induced cell death by activation of the mitochondrial pathway of apoptosis in human colon carcinoma cells [168]. Alternariol and its 9-methyl ether induced cytochrome P450 1A1 and apoptosis in murine heptatoma cells dependent on the aryl hydrocarbon receptor [169]. Other alternariol derivatives such as alternariol 5-*O*-sulfate (**158**), alternariol 9-methyl ether (**159**), 3'-hydroxyalternariol (**161**), altenuene (**162**), 4'-epialtenuene (**164**) and dehydroaltenusin (**156**) were also screened to be cytotoxic [84]. Dehydroaltenusin (**156**), isolated from *A. tenuis*, was found to be a specific inhibitor of eukaryotic DNA polymerase α to show its strong cytotoxic activity on tumor cells [83,170].

Some screened *Alternaria* anthraquinones displayed cytotoxic activity. Demethylmacrosporin (**175**) was cytotoxic to Hela and KB cells with IC_50_ values of 7.3 μg/mL and 8.6 μg/mL, respectively [32]. Altersolanol C (**187**) was also screened to show cytotoxic activity on a few tumor cells [90]. A few bianthraquinones including alterporriols A/B (**195**), C (**196**), D/E (**197**), F (**198**), K (**199**), L (**200**), and P (**204**) showed strong cytotoxic activity on a few tumor cells [32,90,100,171]. Alterporriol L (**200**), a bianthraquinone derivative isolated from a marine fungus *Alternaria* sp. ZJ9-6B, inhibited the growth and proliferation of the MDA-MB-435 breast cancer cells through destroying the mitochondria [171].

Some *Alternaria* phenolic metabolites also have cytotoxic activity. Alterlactone (**244**) from *Alternaria* sp. was toxic on L5178Y mouse lymphoma cells [84]. Alternethanoxins A (**245**) and B (**246**) from *A. sonchi* displayed growth inhibitory activity on six cancer cell lines [172]. Both 6-(3',3'-dimethylallyloxy)-4-methoxy-5-methylphthalide (**253**) and 5-(3',3'-dimethylallyloxy)-7-methoxy-6-methylphthalide (**255**) were proved to have anti-tumor promoting activity [7]. 5-(3',3'-dimethylallyloxy)-7-methoxy-6-methylphthalide (**255**) had the cytotoxicity on Hela cells and KB cells with IC_50_ values as 36.0 μg/mL and 14.0 μg/mL, respectively. Porriolide (**256**) had the cytotoxicity on KB cells with IC_50_ value as 59.0 μg/mL [32]. Depudecin (**257**), an eleven-carbon linear polyketide from *A. brassicicola*, is an inhibitor of histone deacetylase (HDAC) to show its potential in cancer therapy [9].

### 3.3. Antimicrobial Activity

Three diketopiperazine dipeptides namely cyclo-[l-Leu-*trans*-4-hydroxy-L-Pro-] (**37**), cyclo-(l-Phe-*trans*-4-hydroxy-l-Pro-) (**44**), and cyclo-(l-Ala-*trans*-4-hydroxy-l-Pro) (**45**) extracted from broth culture of the grapevine endophyte *A. alternata* showed effectiveness by inhibiting sporulation of the pathogen *Plasmopara viticola* at concentrations of 10^−3^, 10^−4^, 10^−5^ and 10^−6^ mol/L. This indicated that endophytic fungus *A. alternata* can be used as biocontrol agent to control fungal disease in grapevine cultivation [44]. Cyclo-(Phe-Ser-) (**36**) from *Alternaria* sp. FL25 showed antifungal activity on *Fusarium graminearum*, *F. oxysporum* f.sp. *cucumernum*, *F. oxysporum* f.sp. *neverum*, *Phytophthora capsici*, *Colletotrichum gloesporioides* with MICs from 6.25 to 25.00 μg/mL [43]. Tenuazonic acid (**15**) was found to be an active compound in *A. alternata* against *Mycobacterium tuberculosis* H37Rv with MIC value of 250 μg/mL. This compound was thought as a promising antitubercular principle [28]. Other nitrogen-containing metabolites with antimicrobial activity included altersetin (**12**), pyrophen (**14**), tenuazonic acid (**15**) and brassicicolin A (**58**) [21,23,28,50,51,159].

Helvolic acid (**117**) from *Alternaria* sp. FL25, an endophytic fungus in *Ficus carica*, showed the strong antifungal activity on all tested phytopathogenic fungi (*Alternaria alternata*, *A. brassicae*, *Botrytis cinerea*, *Colletotrichum gloesporioides*, *Fusarium graminearum*, *F. oxysporum*, *F. oxysporum* f.sp. *fragariae*, *F. oxysporum* f.sp. *niveum*, *Phytophthora capsici*, *Valsa mali*) with MICs of 1.56–12.50 μg/mL [43].

Herbarin A (**132**) and altechromone A (**151**) from *A. brassicicola* ML-P08 exhibited antimicrobial activity on *Trichophyton rubrum*, *Candida albicans*, *Apergillus niger*, *Bacillus subtilis*, *Escherichia coli*, *Pseudomonas fluorescens* with MICs ranged from 1.8 to 62.5 μg/mL [55]. Rubrofusarin B (**167**) from *A. alternata* showed antifungal activity on *Candida albicans* [23].

Some anthraquinone metabolites, e.g., macrosporin (**174**), hydroxybostrycin (**184**), altersolanol A (**185**), altersolanol B (**186**), altersolanol C (**187**), altersolanol G (**191**), and alterporriol C (**196**) from *A. solani* and *Alternaria* sp. showed antibacterial activity on *Bacillus subtilis*, *Escherichia coli*, *Micrococcus luteus*, *Pseudomonas aeruginosa*, *Staphylococcus albus*, *Staphylococcus aureus*, *Vibrio parahemolyticus* [90,94,97]. Two perylenequnones alterperylenol (**207**) and dihydroalterperylenol (**209**) from *Alternaria* sp. had antifungal activity on *Valsa ceratosperma* [101].

Altenusin (**241**) and porric acid D (**243**) from *Alternaria* sp. showed inhibitory activity against *Staphyloccus aureus* with MICs of 100 μg/mL and 25 μg/mL, respectively [123]. (4S)-α,β-Dehydrocurvularin (**250**) from *Alternaria* sp. showed inhibitory activity on appressorium formation of *Magnaporthe oryzae* [86], and antibacterial activity on *Proteus vulgaris* and *Salmonella typhimurium* with MICs as 25 μg/mL [129].

### 3.4. Other Bioactivities

Altenusin (**241**) isolated from the endophytic fungus *Alternaria* sp. (UFMGCB55) in *Trixis vauthieri* (Compositae) was screened to show inhibitory activity on trypanothione reductase (TR), which is an enzyme involved in the protection of the parasitic *Trypanosoma* and *Leishmania* species against oxidative stress, and has been considered to be a validated drug target. Altenusin (**241**) had an IC_50_ value of 4.3 μM in the TR assay [122].

The association of mycotoxins from *Alternaria* fungi with human and animal health is not a recent phenomenon. *Alternaria* toxins have been linked to a variety of adverse effects (*i.e.*, genotoxic, mutagenic, and carcinogenic) on human and animal health [173]. Tenuazonic acid (**15**) has been studied in detail for its toxicity to several animal species, e.g., mice, chickens, dogs. In dogs, it caused haemorrhages in several organs at daily doses of 10 mg/kg, and in chickens, sub-acute toxicity was observed with 10 mg/kg in the feed. In particular, increasing tenuazonic acid in chicken feed from sublethal to lethal levels progressively reduced feed efficiency, suppressed weight gain and increased internal haemorrhaging. Tenuazonc acid (**15**) is more toxic than AOH (**157**), AME (**159**) and ALT (**162**) [25,167]

There were a few reports about the toxicity of *Alternaria* metabolites on brine shrimp (*Artemia salina* L.) [23,107,174,175]. The LC_50_ values of tenuazonic acid (**15**), alternariol (**157**), altenuene (**162**) and altertoxin-I (**212**) were 75, 100, 375 and 200 μg/mL, respectively, to brine shrimp larvae by using the disk method of inoculation and an exposure period of 18 h [175]. Tenuazonic acid (**15**), alternariol (**157**), alternariol 9-methyl ether (**159**), altenuene (**162**), altertoxin I (**212**) were also verified to toxic to brine shrimp by other investigators [27,174,175]. Six naphthopyranones, namely rubrofusarin B (**167**), fonsecin (**168**), aurasperone A (**170**), aurasperone B (**171**), aurasperone C (**172**) and aurasperone F (**173**) from the marine-derived fungal strain *A. alternata* were screened to show inhibitory activity on brine shrimp (*Artemia salina* L.) at 10 μg/mL [23].

## 4. Conclusions and Future Perspectives

We just clarified one part of metabolites from the known *Alternaria* fungi. The rest of metabolties in *Alternaria* species need to be investigated in detail. In fact, many other *Alternaria* species remain unexplored for their metabolites. In most cases, both the biological activities and modes of action of the metabolites from *Alternaria* fungi have been studied very primarily. The structure-activity relationship has been established only for a few classes of *Alternaria* metabolites. This review mainly focused on the metabolites with low molecular weight from *Alternaria* fungi. Bioactive proteins, saccharides and glycoproteins are also important metabolites. Typical examples included a lipase from *A. brassicicola* [176], an endopolygalacturonase from the rough lemon pathotype of *A. alternata* [177], a protein elicitor (Hrip1) from *A. tenuissima* [178], and a polyketide synthase from *A. alternata* [179]. Some bioactive saccharides and glycoproteins have also been isolated such as β-1,3-, 1,6-oligoglucan elicitor from *A. alternata* [180] and a glycoprotein elicitor from *A. tenuissima* [181].

The potential applications of *Alternaria* metabolites as antitumor agents, herbicides, and antimicrobials as well as other promising bioactivities have led to considerable interest within the pharmaceutical community. Chemical syntheses have been achieved for a few bioactive metabolites such as AAL-toxin TA_1_ (**1**) [182], maculosin (**43**) [183], AM-toxin I (**46**) [184], alternariol (**157**) [185], alternariol 9-methyl ether (**159**) [185], altenuene (**162**) [186], isoaltenuene (**163**) [186], neoaltenuene (**166**) [187], altertoxin III (**218**) [188], zinniol (**226**) [189], altenusin (**241**) [190] and alterlactone (**244**) [190].

In recent years, more and more *Alternaria* fungi have been isolated as plant endophytic fungi from which large amounts of bioactive compounds have been structurally characterized. Another approach is to discovery novel bioactive compounds from the *Alternaria* fungi isolated from marine organisms. These *Alternaria* fungi could be the rich sources of biologically active compounds that are indispensable for medicinal and agricultural applications [191].

After comprehensive understanding of biosynthetic pathways of some *Alternaria* metabolites in the next few years, we can effectively not only increase yields of the bioactive metabolites, but also prohibit biosynthesis of some toxic metabolites (*i.e*., phytotoxins and mycotoxins) by treatment with some special fungicides.
